# A Preliminary Study of the Effect of Quercetin on Cytotoxicity, Apoptosis, and Stress Responses in Glioblastoma Cell Lines

**DOI:** 10.3390/ijms23031345

**Published:** 2022-01-25

**Authors:** Magdalena Kusaczuk, Rafał Krętowski, Monika Naumowicz, Anna Stypułkowska, Marzanna Cechowska-Pasko

**Affiliations:** 1 Department of Pharmaceutical Biochemistry, Medical University of Bialystok, 15-222 Bialystok, Poland; r.kretowski@umb.edu.pl (R.K.); anna.stypulkowska@umb.edu.pl (A.S.); mapasko@gmail.com (M.C.-P.); 2 Department of Physical Chemistry, Faculty of Chemistry, University of Bialystok, 15-245 Bialystok, Poland; monikan@uwb.edu.pl

**Keywords:** apoptosis, ER stress, glioblastoma, necrosis, oxidative stress, quercetin

## Abstract

A growing body of evidence indicates that dietary polyphenols show protective effects against various cancers. However, little is known yet about their activity in brain tumors. Here we investigated the interaction of dietary flavonoid quercetin (QCT) with the human glioblastoma A172 and LBC3 cell lines. We demonstrated that QCT evoked cytotoxic effect in both tested cell lines. Microscopic observations, Annexin V-FITC/PI staining, and elevated expression and activity of caspase 3/7 showed that QCT caused predominantly apoptotic death of A172 cells. Further analyses confirmed enhanced ROS generation, deregulated expression of SOD1 and SOD2, depletion of ATP levels, and an overexpression of CHOP, suggesting the activation of oxidative stress and ER stress upon QCT exposure. Finally, elevated expression and activity of caspase 9, indicative of a mitochondrial pathway of apoptosis, was detected. Conversely, in LBC3 cells the pro-apoptotic effect was observed only after 24 h incubation with QCT, and a shift towards necrotic cell death was observed after 48 h of treatment. Altogether, our data indicate that exposure to QCT evoked cell death via activation of intrinsic pathway of apoptosis in A172 cells. These findings suggest that QCT is worth further investigation as a potential pharmacological agent in therapy of brain tumors.

## 1. Introduction

Glioblastoma multiforme (GBM) is considered one of the most frequent and aggressive type of brain tumors with an overall dismal survival rate averaging one year. Despite multimodal therapeutic interventions including surgery, radiotherapy, and adjuvant chemotherapy, this type of cancer still presents poor response to the available treatments [1,2,3,4]. To address the need for new therapeutic strategies and due to the failure of classical chemotherapies, research efforts are now focused on the use of efficient but less toxic therapeutic agents [4].

In the last two decades, natural product-based therapy has gained popularity as an effective and potentially less toxic treatment [4]. In this respect, the field of natural compounds with anticancer properties has currently been explored also in the management of brain malignancies. Preliminary reports on the application of natural products in cancer treatment are promising, with increased data suggesting anti-proliferative effect in cancer cells compared to normal cells [5,6,7]. One such group of compounds with beneficial effects against cancer are flavonoids. Flavonoids are widely distributed in the form of benzo-gamma-pyrone derivatives in flowers and fruits. They are represented by a diverse group of compounds comprised of flavanones, chalcones, flavones, and isoflavones. These substances have been shown to display certain favorable features against GBM, such as the ability to cross the BBB and relatively low toxicity to primary cells [7,8,9,10]. Many of these reports highlight quercetin (QCT) as one of the most potent anticancer compounds. Quercetin (chemically 3,3′,4′,5,7-pentahydroxy flavone) is found in a variety of plant-based foods such as red onions, broccoli, capers, parsley, apples, tea, red grapes, and a number of berries [11].

In many in vitro and in vivo models of cancer, QCT has been extensively studied as a chemopreventive agent exerting anti-inflammatory and antioxidant activity (mainly through scavenging of the reactive oxygen/nitrogen species) [7]. Additionally, in certain types of cancer cells QCT has been reported to interfere with cell proliferation, differentiation, and survival via targeting various key molecules responsible for tumor cell growth. Thus, QCT has been demonstrated to interact with molecules such as p53, p21, Ras, and mediators of PI3K/AKT pathway [12,13]. To date, several reports illustrating the utility of QCT for brain tumor treatment have also been published [8,14,15,16]. Specific to GBM, Michaud-Levesque et al., showed that quercetin significantly decreased the IL-6-mediated STAT3 activation in U87 and T98G cell lines [11]. Moreover, other reports demonstrated efficacy of QCT in glioblastoma treatment occurring through inhibition of the PI3K/AKT pathway [13], reduction of X-linked inhibitor of apoptosis protein (XIAP) expression [17], G2-dependent cell cycle arrest [8], modulation of intracellular pH (pHi), as well as regulation of matrix metalloproteinases (MMPs) -2/-9, and vascular endothelial growth factor (VEGF) expression [7,18]. Notably, QCT has also been shown to induce mitochondria mediated apoptosis in the *p53*-mutant GBM cell line U373MG [15], and increase the sensitivity of U87 and U251 cells to temozolomide by suppressing expression of the heat shock protein 27 (HSP27) [19]. Interestingly, very limited number of research investigated the influence of cellular stresses on apoptotic cell death in QCT-treated glioblastoma cells. As such, Jang et al. demonstrated that stimulation with QCT contributed to the death of T98G cells through ER stress, Ca^2+^ imbalance, and oxidative stress (however, only when co-treated with chloroquine) [5]. In this respect, further exploration of the role of stress responses in QCT-mediated apoptosis of GBM cells warrants further investigation.

Despite the vast majority of data reporting apoptosis as the predominant mode of action of QCT [15,16,20,21,22,23], several studies have demonstrated necrotic cell death as cellular phenomenon activated in GBM cells after QCT stimulation [16,24,25]. Notably, the QCT-derived effects in GBM seem to be highly dependent on the cell type. The combined application of tumor necrosis factor-related apoptosis-inducing ligand (TRAIL) and quercetin strongly reduced viability of U251, U87MG, LN229, and A172 cells but failed to inhibit proliferation of U373MG cells [20]. Likewise, C6 cells stimulated simultaneously with resveratrol and QCT showed marked increase in number of inviable cells in comparison to cells exposed to each compound separately, indicating an additive effect of the co-treatment. Interestingly, no such effect was observed in other tested cell lines: U87MG, U138MG nor GL261 [10]. The reasons of this cell line-specificity may be various, from molecular signature of cells to the composition of the cell membrane. The first one may be dependent on the mutation status of genes such as *Pten* or *p53*, and the latter has been recently studied as determinant of drug penetration (a summary of QCT effects combined with a status of mutations in crucial cancer progression-related genes in several GBM cell lines is presented in Table 1).

Lately, preliminary data showing the importance of membrane component in drug sensitivity of cancer cells has been reported [35,36,37,38,39,40,41]. Phenolic compounds tend to change their dissociation status under certain pH, which may therefore mediate different affinity of polyphenols to the functional groups exposed on the surface of cell membranes and, at least partially, condition intracellular penetration potential of a drug [35,36,42]. In this respect, better understanding of the multidirectional aspects of QCT functioning in GBM may contribute to the development of more efficient therapeutic strategies based on the application of natural compounds.

## 2. Results

### 2.1. The Effect of QCT on Cell Viability

**Figure 1:**
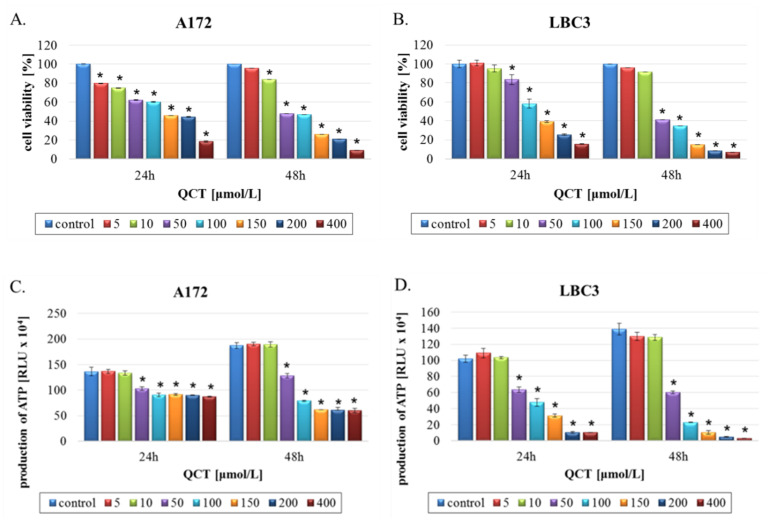
The viability of glioblastoma A172 and LBC3 cells treated with QCT. Results of the MTT test are shown for A172 (**A**) cells and LBC3 (**B**) cells. Results of the CellTiter-Glo assay are presented for A172 cells (**C**) and LBC3 cells (**D**). Cells were incubated with various concentrations of QCT 5–400 μmol/L for 24 and 48 h. The results represent means for pooled triplicate values from three independent experiments. * *p* < 0.05.

Antiproliferative effects of QCT on A172 and LBC3 glioblastoma cells were assessed using MTT test and luminescent CellTiter-Glo assay. Cells were exposed to increasing concentrations (5–400 μmol/L) of QCT for 24 h and 48 h. QCT stimulation resulted in dose- and time-dependent reductions in cell viability for both cell lines (Figure 1). Results of the MTT analysis showed that QCT had high ability to limit cell proliferation of either A172 (Figure 1A) and LBC3 (Figure 1B) cells. In both cases, QCT decreased cell viability to a similar extent after 24 h and 48 h of treatment, with the most pronounced decrease in viability starting at a concentration of 50 µmol/L QCT. A172 cells stimulated with 50 μmol/L QCT were 40.73% ± 4.91% unviable after 24 h, and 53.47% ± 5.36% unviable after 48 h. At 100 μmol/L, these values were 41.77% ± 4.35% vs. 54.45% ± 5.18% for 24 h and 48 h, respectively (Figure 1A). Finally, only ≈10% of viable A172 cells was reached at the highest 400 μmol/L concentration of QCT after 48h (Figure 1A). In comparison, LBC3 cells exposed to the same concentrations of QCT showed even more pronounced cytotoxic effects, approaching nearly 95% unviable cells, at the highest concentration after 48 h of treatment. In this case, time-dependent loss of viability was most apparent (Figure 1B). The IC_50_ values calculated after 48 h of incubation were 58.5 and 41.37 μmol/L for A172 and LBC3 cells respectively. Based on the MTT results, we chose to proceed with two different concentrations of QCT for further examination. Doses of 50 μmol/L and 100 μmol/L were chosen in order to examine cytotoxic concentrations of QCT.

Furthermore, to confirm mitochondrial toxicity of QCT evaluation of the ATP levels was performed. These results were in general agreement with the MTT-based cytotoxicity evaluation, presenting dose- and time-dependent decrease in ATP production in QCT-stimulated GBM cells. However, more pronounced drop in the ATP levels was observed in LBC3 cells in comparison to A172 cell line, which may suggest stronger mitochondria-damaging effect of QCT in LBC3 cells (Figure 1C,D).

### 2.2. The Effect of QCT on Cell Death

**Figure 2:**
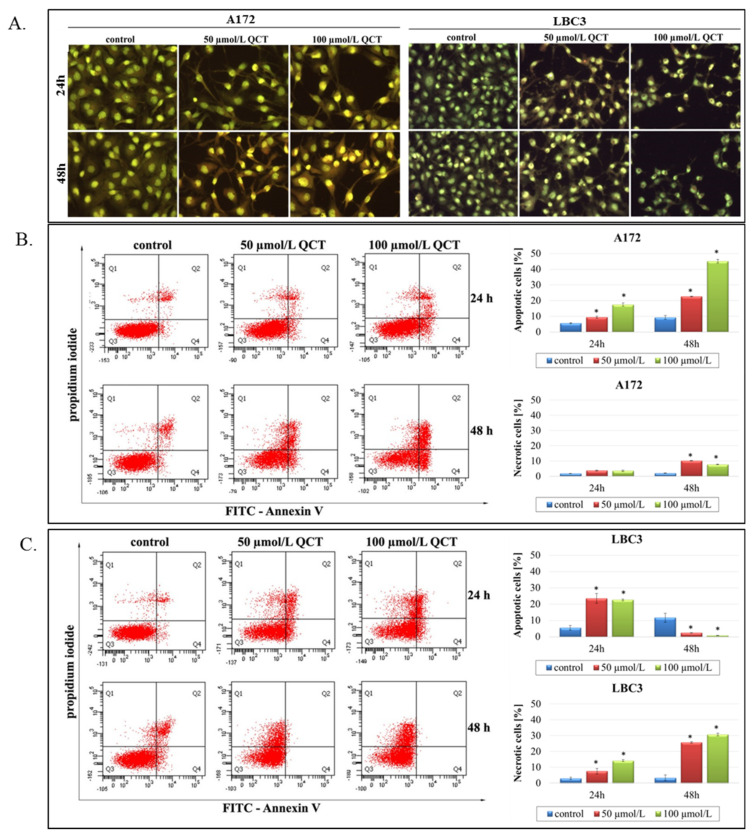
The effect of QCT on apoptosis of glioblastoma cells. A172 and LBC3 cells were incubated with 50 μmol/L or 100 μmol/L of QCT for 24 h and 48 h. Phenotypic characteristics of A172 and LBC3 (**A**) cells are presented. Morphological effects induced by QCT treatment were evaluated by AO–EtBr staining and visualized by fluorescence microscopy (magnification 200×). Flow-cytometry analysis of A172 and LBC3 cells incubated with 50 μmol/L and 100 μmol/L QCT for 24 h and 48 h (**B**,**C**). Representative FACS images of cells subjected to annexin V–FITC/propidium iodide staining, together with the calculated percentages of apoptotic and necrotic cells for A172 (**A**) and LBC3 (**C**) cells are presented. Mean ± SD from three independent experiments are shown. * *p* < 0.05.

Microscopic observations were carried out to determine whether the reduced viability of QCT-treated A172 and LBC3 cells was accompanied by alterations in cellular morphology and increased cell death. Staining of both cell lines with AO–EtBr revealed dose- and time-dependent decrease in cell density and an increased number of cells with morphologically changed cytoplasm and nuclei, indicative of augmented cell death (Figure 2A).

To confirm these microscopic results, we performed flow cytometry analysis (Figure 2B–G). Treatment of A172 cells with QCT at 50 μmol/L or 100 μmol/L for 24 h resulted in markedly elevated levels of apoptotic cells (9.43% and 17.47%, respectively) in comparison to control (Figure 2B). After 48 h, the proapoptotic effect became more pronounced, with nearly 23% and 45% of cells undergoing apoptosis at 50 μmol/L and 100 μmol/L dose of QCT, respectively. Necrosis was a marginal phenomenon in A172 cells (Figure 2B). In contrast, in LBC3 cells apoptotic cell death was observed only in cells incubated with QCT for 24 h, but not 48 h (Figure 2C). Here, necrosis was a dominant way of cell elimination, reaching up to 30% of necrotic cells in 100 μmol/L after 48 h (Figure 2C). Our results suggest that QCT-dependent induction of apoptosis in LBC3 cells may be followed by necrosis occurring over a longer incubation time.

**Figure 3:**
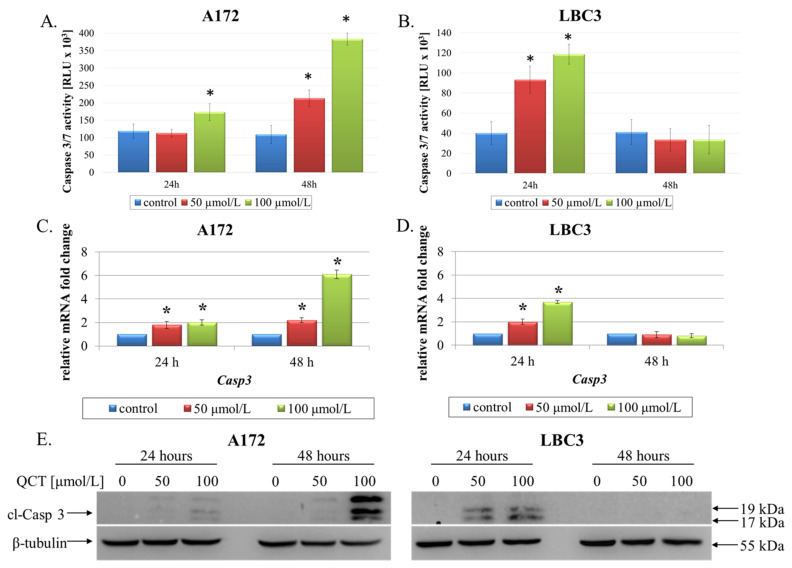
The effect of QCT on activation of caspase 3. Caspase 3/7 activity in A172 (**A**) and LBC3 (**B**) cells exposed to 50 μmol/L or 100 μmol/L of QCT for 24 h and 48 h. RT-qPCR analysis of *Casp3* in A172 (**C**) and LBC3 (**D**) cells. Results shown as relative fold change in mRNA expression in comparison to untreated controls, where expression level was set as 1. Western blot analysis of cleaved-Casp 3 expression in A172 and LBC3 cells (**E**). Mean ± SD from three independent experiments are shown. * *p* < 0.05.

To further explore the mechanism of cell death in A172 and LBC3 cell lines, we assayed the cells for evidence of caspase activation. In A172 cells incubated for 24 h significant elevation in caspase 3/7 activity was observed only in cells stimulated with 100 μmol/L QCT (Figure 3A). However, after 48 h of QCT incubation at either 50 μmol/L or 100 μmol/L, we observed nearly twofold and fourfold increases in caspase 3/7 activity (Figure 3A). These results were additionally confirmed on transcriptomic level, by evaluating mRNA expression of *Casp3* (Figure 3C), and proteomic level by western blot assay of cleaved-caspase 3 (cl-Casp 3) (Figure 3E). Accordingly, elevated activity of caspase 3/7 in LBC3 cells was only detected in cells exposed to QCT for 24 h but not 48 h (Figure 3B). These results were also reflected in RT-qPCR and western blot analyses, showing marked up-regulation of *Casp3* and overexpression of cl-Casp 3 in cells treated with 50 μmol/L and 100 μmol/L QCT for 24 h but not 48 h (Figure 3D,E).

### 2.3. The Effect of QCT on ER Stress

**Figure 4:**
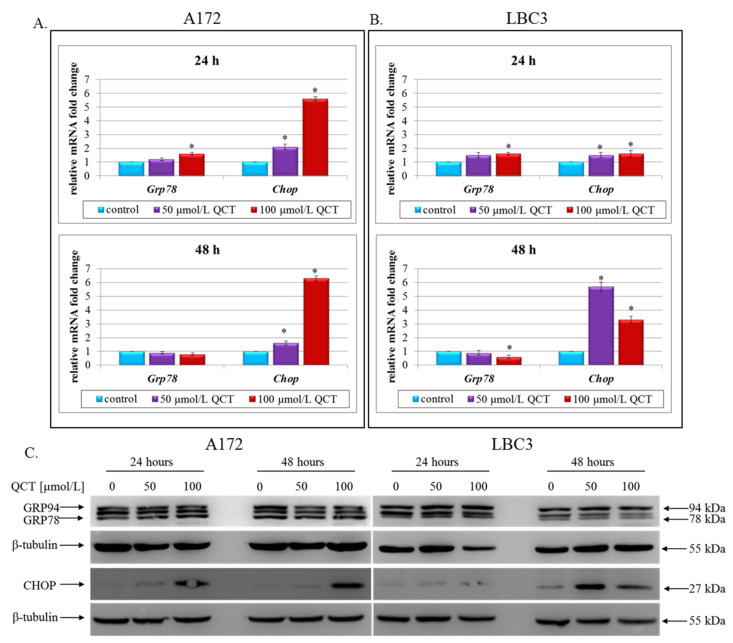
The effect of QCT on ER stress in glioblastoma cells. RT-qPCR analysis of *Grp78* and *Chop* expressions in A172 (**A**) and LBC3 (**B**) cells. Cells were incubated with 50 or 100 μmol/L of QCT and total RNA was extracted from cells cultured for 24 h and 48 h. Results shown as relative fold change in mRNA expression in comparison to untreated controls, where expression level was set as 1. Western blot analysis of GRP78, GRP94, and CHOP expressions in A172 and LBC3 cells (**C**). Mean ± SD from three independent experiments are shown. * *p* < 0.05.

To determine whether QCT induced ER stress in A172 and LBC3 cells, two markers of this phenomenon (the proapoptotic transcription factor CHOP and molecular chaperone GRP78) were assayed (Figure 4). RT-qPCR analysis of QCT-stimulated A172 cells revealed only partially altered expressions of *Grp78* and *Chop* at the mRNA level (Figure 4A). Interestingly, regardless of the time of incubation and the applied dosage of QCT *Grp78* did not show significantly different expression from this observed in the control cells, except for slight but relevant up-regulation in 100 μmol/L QCT for 24 h (Figure 4A). In contrast, *Chop* showed marked upregulation at 50 μmol/L and 100 μmol/L QCT at both incubation times in comparison to control (Figure 4A). These results were also confirmed at the protein level (Figure 4C). Immunoblot detection of GRP78 using anti-KDEL antibody, which simultaneously bound to another ER-resident chaperone—GRP94, showed almost unaffected expression of ER chaperones in QCT-treated cells in relation to control counterparts (Figure 4C). As opposed to the unchanged GRP78/GRP94 levels, CHOP was markedly overexpressed in A172 cells exposed to QCT (Figure 4C), confirming the results of RT-qPCR analysis (Figure 4A). Notably, distinct results were observed for LBC3 cell line. Here, after 48 h of incubation with QCT the cells demonstrated significant down-regulation of *Grp78* (Figure 4B), which was further confirmed at the protein level (Figure 4C). Also, *Chop* expression was significantly up-regulated in cells exposed to QCT (Figure 4B). The results demonstrated greater than quintuple up-regulation of *Chop* transcript in cells exposed to 50 μmol/L QCT, and over triple up-regulation in cells stimulated with 100 μmol/L QCT after 48 h (Figure 4B). These results were also mirrored by immunoblot evaluation (Figure 4C). These data suggest that treatment with QCT may disrupt ER homeostasis and initiate an ER-stress state in both A172 and LBC3 cells.

### 2.4. The Effect of QCT on Oxidative Stress

**Figure 5:**
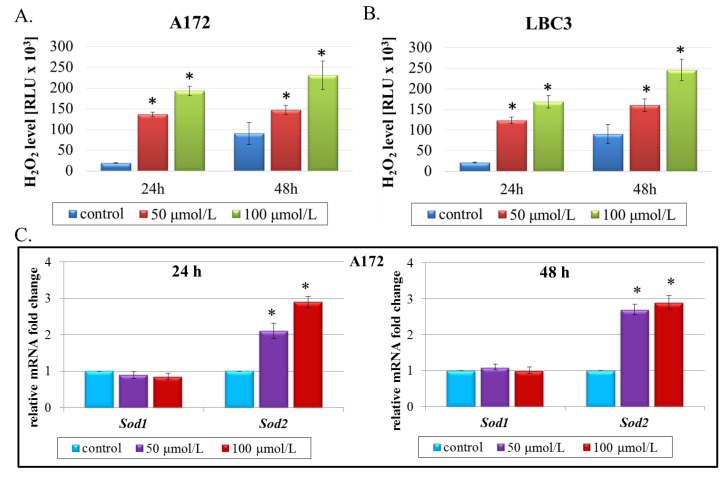

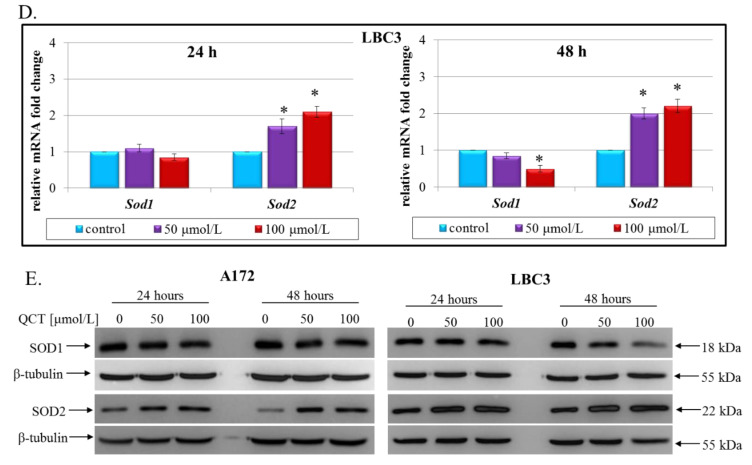
The effect of QCT on oxidative stress in glioblastoma cells. A172 and LBC3 cells were incubated with 50 or 100 μmol/L of QCT for 24 h or 48 h. ROS generation in A172 (**A**) and LBC3 (**B**) cells. RT-qPCR analysis of antioxidant-enzyme genes *Sod1* and *Sod2* in A172 (**C**) and LBC3 (**D**) cell lines. Results shown as relative fold change in mRNA expression in comparison to untreated controls, where expression level was set as 1. Western blot analysis of SOD1 and SOD2 expressions in A172 and LBC3 cells (**E**). Mean ± SD from three independent experiments are shown. * *p* < 0.05.

To gain further insight into the cytotoxicity of QCT in A172 and LBC3 cells, we checked if the oxidative stress was evoked upon QCT treatment. In this respect, ROS levels were examined. Although a variety of ROS is generated in cell cultures including singlet oxygen, hydroxyl radical, hydrogen peroxide, and superoxide exist, many of these are converted into H_2_O_2_. Thus, measurement of this molecule is a convenient proxy for assaying overall ROS levels in cells. Our results demonstrated that QCT-induced intracellular ROS generation changed in a dose- and time-dependent manner in both cell lines. As presented in Figure 5A, in A172 cells ROS production was significantly increased after exposure to 50 μmol/L as well as 100 μmol/L QCT at both incubation times. Similar results were observed for LBC3 cells (Figure 5B). Overproduction of ROS disturbs the balance between oxidative and antioxidative systems, resulting in reduced antioxidative capacity. To determine whether enhanced ROS production was accompanied by the deregulated expression of key antioxidative enzymes, real-time qPCR analysis of *Sod1* and *Sod2* was performed (Figure 5C,D). These results were additionally validated by immunoblot detection of SOD1 and SOD2 proteins (Figure 5E). Indeed, we noticed markedly disrupted expression of the analyzed genes. Of note, in A172 cells *Sod1* expression was unbiased in QCT-treated cells in comparison to control, whereas *Sod2* was significantly upregulated especially after 48-h exposure to QCT (nearly double increase, independently of QCT concentration) (Figure 5C). The results of RT-qPCR analysis were also in accordance with SOD1 and SOD2 evaluation on the protein level (Figure 5E). Slightly different results were obtained in case of LBC3 cells. Here, significant attenuation of SOD1 expression in cells stimulated with 100 μmol/L QCT for 48 h was observed at both mRNA and protein levels (Figure 5D,E), and only slight, yet significant alterations in SOD2 expression in comparison to control were detected (Figure 5D,E).

**Figure 6:**
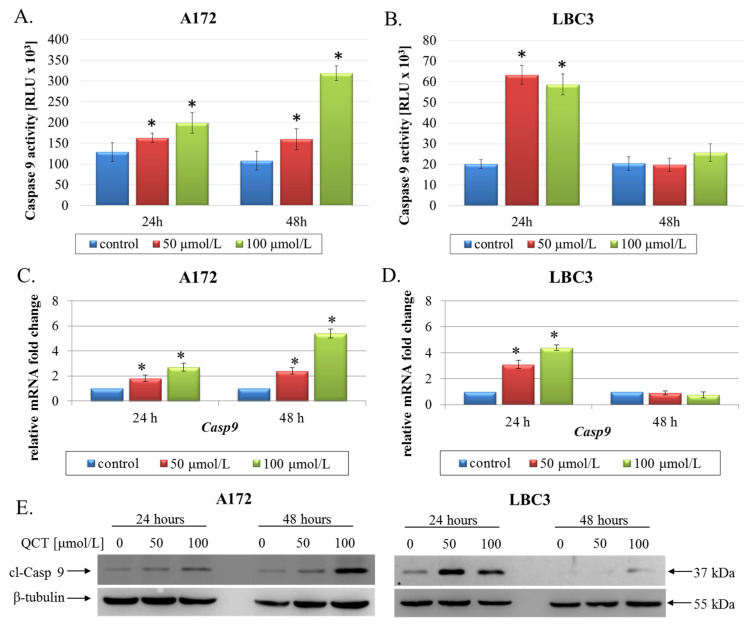
The effect of QCT on activation of caspase 9. Caspase 9 activity in A172 (**A**) and LBC3 (**B**) cells exposed to 50 μmol/L or 100 μmol/L of QCT for 24 h and 48 h. RT-qPCR analysis of *Casp9* in A172 (**C**) and LBC3 (**D**) cells. Results shown as relative fold change in mRNA expression in comparison to untreated controls, where expression level was set as 1. Western blot analysis of cl-Casp 9 expression in A172 and LBC3 cells (**E**). Mean ± SD from three independent experiments are shown. * *p* < 0.05.

Next, to determine whether these perturbations induced mitochondrial pathway of apoptosis, we evaluated the expression and activity of caspase 9, an indicator of mitochondria-dependent cell death. We demonstrated that 24 h exposure to QCT was sufficient to increase the expression and augment the activity of caspase 9 in both tested cell lines (Figure 6A–E). However, after 48 h of treatment, levels of caspase 9 expression and activity were markedly elevated only in A172 cells (Figure 6A,C,E) but not in LBC3 cells (Figure 6B,D,E). These results seem to be in line with the mostly necrotic LBC3 cells identified after 48 h of QCT treatment, and mostly apoptotic A172 cells occurring at both incubation times, and LBC3 cells after 24 h of QCT stimulation.

Altogether, these data indicate that QCT induced oxidative stress via ROS overproduction, followed by disruption of the oxidant–antioxidant system balance, impaired mitochondria function, and finally activation of the intrinsic apoptosis pathway.

### 2.5. The Effect of QCT on the Surface Charge Density of Cellular Membranes

Recently, it has become evident that certain characteristics of cancer cell membranes ought to be highlighted in order to improve drug development. The parameters such as membrane charge, membrane surface packing, membrane lipid composition, as well as membrane asymmetry and fluidity seem to have a high impact on potential drug-membrane interactions. Given this, studies of these interactions arise as important tools to better understand the therapeutic activity of antineoplastic agents and their toxic actions [42]. In this respect, in order to gain deeper insight into the nature of QCT-membrane interactions, measurements of surface charge density of A172 and LBC3 cell membranes subjected to QCT treatment have been done.

**Figure 7:**
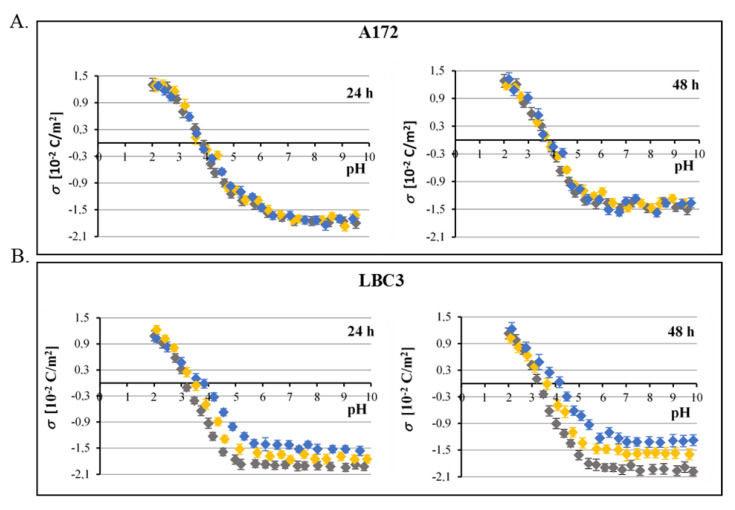
The effect of QCT on surface charge density of biological membranes of glioblastoma cells. pH dependence of surface charge density of A172 (**A**) and LBC3 (**B**) cell membranes as a function of 0 (♦), 50 (♦), and 100 (♦) μmol/L QCT after 24 h and 48 h of treatment. The results represent mean values from three independent experiments run in triplicate.

The pH dependencies of the surface charge of the A172 and LBC3 membranes are plotted in Figure 7A,B respectively. Data are presented for control cells as well as cells treated with 50 and 100 μmol/L of QCT. The obtained dependencies are of similar shape for all analyzed cell membranes. The decrease in pH values was followed by an increase in positive surface charge density, albeit only up to a certain point. Conversely, along with an increase in pH values, the negative charge of the membranes increased until the plateau was achieved. The data presented in Figure 7A demonstrate that for A172 glioblastoma cells treated with both concentrations of QCT for 24 h and 48 h, the value of surface charge density is independent on the dose and time of treatment. Furthermore, no noticeable changes were observed in the isoelectric point values of analyzed GBM cell membranes cultured with QCT. This allowed us to conclude that in A172 cells, QCT does not adsorb on the membrane surface and easily passes through the lipid bilayer within a wide range of pH to act intracellularly. It is also noteworthy that at low pH values, no visible changes in the surface charge density values were found in membranes of LBC3 cells incubated with QCT (Figure 7B). However, at high pH values the presence of QCT resulted in decrease in the negative charge of cell membranes in comparison to the untreated control cells. This effect was evidently dose and time dependent. Moreover, the isoelectric point of LBC3 cell membranes treated with QCT shifted to higher pH values compared to untreated control (from pH~3.1 to pH~3.8 and pH~4.2 after 24 h and 48 h respectively). These alterations may correspond with the changes in the functional group composition of glioblastoma cell membranes, which can be due to the appearance or disappearance of new chemical moieties resulting from the reactions with QCT. Nevertheless, since in LBC3 cells the main cellular effect caused by QCT was necrosis, these assumptions should be treated with caution. Necrosis refers to the progressive loss of plasma membrane integrity along with a decrease in cytosolic pH and an influx of ions across the membrane, which may also affect surface charge density of cell membranes. Therefore, these results warrant further exploration in order to be able to make definitive conclusions on drug-membrane interactions. Consequently, physicochemical investigations should be joined by comprehensive biochemical analysis in attempt to identify the most promising drug candidates in the future.

## 3. Discussion

**Figure 8:**
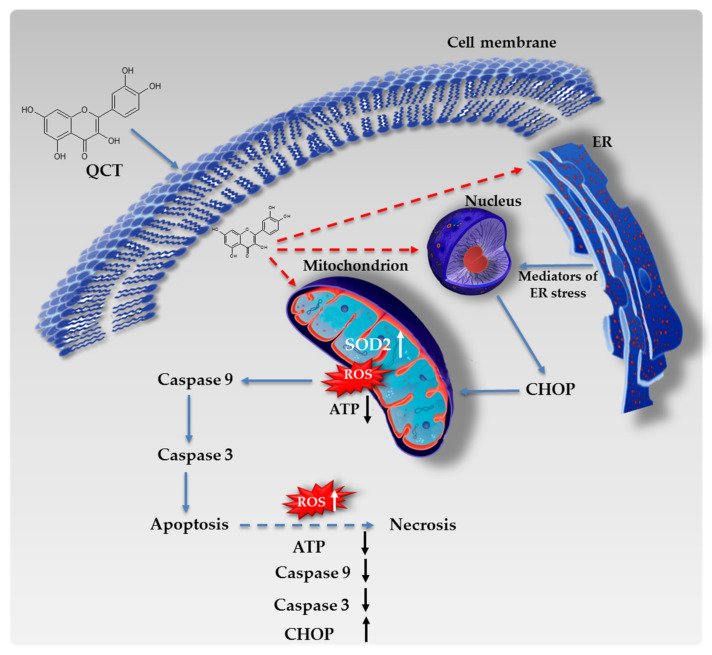
Tentative model of quercetin mode of action in GBM cells. Dashed red arrows indicate probable organelles targeted by QCT in glioblastoma cells; dashed blue arrow indicates tentative possibility of the switch between apoptosis and necrosis in GBM cells.

Glioblastoma is a type of cancer that still poses a therapeutic challenge with poor overall prognosis [1,37,43]. Given this, new therapeutic approaches aiming at inhibiting cancer cell growth remain an area of active investigation. Currently, it is widely recognized that natural compounds represent a class of promising anticancer agents capable of counteracting tumor development while simultaneously being relatively non-toxic to normal cells [7,8,9,10]. Various phytochemicals have already been tested in countless in vitro and in vivo studies presenting promising results against many types of malignancies [5,6,7,12,14]. One such compound subjected to thorough examination in cancer research is quercetin (QCT). Quercetin is a polyphenolic secondary metabolite that belongs to the flavanol class of flavonoids [21]. Dependent on the time of exposure and the dosage, quercetin may act both ways (i.e., as an antioxidant scavenging free radicals and preventing apoptosis and as a pro-oxidative agent decreasing levels of glutamate-stimulating hormones (GSH) and facilitating apoptosis) [44]. As opposed to normal cells, the cytotoxic effect of QCT can be beneficial for killing tumor cells and controlling cancer progression. However, although considerable number of studies exploring the influence of QCT on various cancer cell lines exists, the molecular mechanisms initiated during QCT-mediated cellular responses in GBM are as of yet insufficiently understood. One such mechanism might be connected with the regulation of gene expression. However, the mechanism in which QCT modulates transcription remains obscure. Preliminary reports suggest accumulation of QCT in nuclear structures and binding to the DNA or genome-associated proteins to acts as a cis-regulatory transcription factor [45,46] (Figure 8). Nevertheless, the exact molecular mode of action of this flavonoid remains to be clarified.

To date, quercetin has been demonstrated to cause cell cycle arrest, induce autophagy, reduce cell migration and angiogenesis, decrease mitochondrial membrane potential, and evoke apoptosis in glioblastoma cells [7]. Moreover, preliminary reports suggest that QCT is capable of crossing the blood–brain barrier, emerging as potentially attractive agent for GBM treatment [47,48]. On these premises, we investigated the cytotoxic effect of QCT on glioblastoma A172 and LBC3 cell lines. In our study, we demonstrated that treatment with QCT resulted in cytotoxic effect in both tested cell lines. However, further exploration of this phenomenon showed significant differences in the mechanisms underlying cytotoxicity of QCT. Therefore, we found that A172 cells were eliminated via apoptotic cell death, which was confirmed by cytometric analyses together with overexpression and augmented activity of caspase 3. These findings are in line with previous reports demonstrating apoptosis as leading way of cell elimination in T98G, U373MG, and C6 glioblastoma cell lines subjected to QCT treatment [8,15,16,22]. Moreover, to obtain deeper insight into the mechanism of apoptotic death of A172 cells, we checked if apoptosis might be driven by ER stress and oxidative stress. Indeed, these cells showed increased expression of CHOP—a key ER stress-related pro-apoptotic transcription factor, suggesting engagement of ER stress into the process of apoptosis of A172 cells. Interestingly, expression of cytoprotective molecular chaperones GRP78/GRP94 was not altered by QCT stimulation. This might be potentially related to the constitutive overexpression of the ER molecular chaperones in GBM cells, which is also often linked with enhanced cell survival and augmented drug-resistance [49]. In fact, significantly increased levels of GRP78 in glioblastoma cells and tissues have been already confirmed in several research projects [49,50]. Moreover, since CHOP might trigger the intrinsic apoptotic pathway [51], the crosstalk between ER and oxidative stress may possibly be responsible for the pro-apoptotic effect of QCT in GBM cells (Figure 8).

Next, we evaluated if QCT may act as prooxidant in GBM cells. Accordingly, in A172 cells exposed to QCT treatment we identified elevated levels of ROS, which was accompanied by disrupted homeostasis of antioxidant enzymes, impaired ATP production, and up-regulated expression and activity of caspase 9. This suggests that oxidative stress followed by intrinsic pathway of apoptosis might have been activated in A172 cells subjected to quercetin treatment. These findings are partially in line with previous reports demonstrating engagement of mitochondrial pathway in the pro-apoptotic effect of QCT [15,23,52,53]. Matsuo et al. identified significantly elevated levels of ROS in the presence of certain polyphenols including apigenin, luteolin, flavonol 3-hydroxyflavone, kaempherol, and quercetin in the normal human cells HUVEC and TIG-1 [52]. Liu et al. demonstrated that in the glioblastoma cell line U251, stimulation with QCT resulted in overexpression of pro-apoptotic Bax and decreased expression of anti-apoptotic Bcl-2 [53]. Likewise, Pan et al. reported decreased level of Bcl-2 protein in U87 cells after stimulation with quercetin [23]. In line with this, Kim et al. showed that in the U373 cell line, QCT induced apoptosis by decreasing mitochondrial membrane potential, activating caspase 9 and increasing the level of caspases 3/7 [15]. Interestingly, in our research we found that SOD1 expression was unaffected by quercetin treatment, but the expression of mitochondrial SOD2 was increased in QCT-stimulated A172 cells. However, ROS levels remained elevated. Since SOD2 is a mitochondrial protein, we speculate that its elevated expression is likely due to the enhanced mitochondria efforts to overcome the effects of QCT treatment. However, SOD2 itself might have failed to overcompensate the pro-oxidant effect of QCT resulting in augmented ROS production and oxidative stress (Figure 8). Nonetheless, despite promising results of the preliminary analyses, further examinations need to be performed in order to fully elucidate the mechanisms activated in GBM cells upon QCT treatment. Accordingly, LBC3 cells exhibited considerable level of necrosis occurring mainly after 48 h of treatment with QCT. This observation seems to be in line with several previous reports showing relatively high percentage of necrotic cells in QCT-stimulated oral cancer cell line SCC-9 [54], prostate cancer cell lines DU-145 and PC-3 [55], and glioblastoma cell line T98G [16]. Regarding the lytic nature of necrosis, which is followed by inflammatory processes and release of damage-associated molecular patterns, apoptosis is obviously favorable way of drug-induced elimination of cancer cells [56]. Unfortunately, the mechanisms driving the switch between apoptosis and necrosis are still obscure and various causes are speculated. It has been proposed, that the amount of the energy available to the cell might be involved in the reprogramming or the cell faith [25,57]. Since apoptosis requires induction of many proteins including caspases, sufficient levels of ATP need to be provided. In this respect, mechanisms leading to the decreased ATP production may be responsible for promoting necrosis [25]. Thus, diminished ATP levels together with the decrease in cytosolic pH may result in an influx of ions across the cell membrane and finally cause cell edema [25,57]. Indeed, in contrast to the A172 cells, CellTiter assay and zeta potential measurements identified more severe decrease in ATP level and altered membrane surface charge density in LBC3 rather than in A172 cells. These observations may partially support the hypothesis of cellular ATP level as determinant of apoptosis/necrosis switch. Additionally, very sparse to no activity of caspase 3 nor 9 was detected in LBC3 cells after 48 h of treatment with QCT, where considerable percentage of necrosis occurred. Nevertheless, the engagement of caspases in the process of necrotic cell death cannot be stated clearly without further detailed examinations including application of caspase inhibitors and exploration of other possible mechanisms driven in the absence of caspases [58]. Notably, despite being widely considered as pro-apoptotic protein, CHOP expression was highly increased in LBC3 cells undergoing necrosis. This phenomenon has been already observed in case of other cell types [59,60]. However, the way in which CHOP might be entangled in necrotic cell death remains unknown (Figure 8). Regarding the lack of the detailed and advanced analysis of caspase-dependent/independent signaling pathways activated during necrotic death of LBC3 cells, further studies are necessary to undeniably determine, which mechanisms are activated during the apoptosis/necrosis switch in these cells. Taking into consideration clinical implications of necrogenesis in GBM, the results of our studies might raise certain concerns about using QCT as potential therapeutic or co-therapeutic agent. Data from several clinical investigations revealed that the appearance of necrosis is accompanied by increased tumor malignancy and poor patient prognosis [61,62,63]. However, despite some uncertainties connected with the effectiveness of QCT in clinical use, a case study of a patient suffering from rapidly progressing advanced multifocal GBM showed certain beneficial effects of QCT as a co-treatment with the standard protocol [64]. This underscores the need for well-established alternative therapies to prevent and effectively treat glioblastoma.

## 4. Materials and Methods

### 4.1. Reagents

DMEM containing glucose at 4.5 mg/mL (25 mmol/L) with GlutaMax, streptomycin, penicillin, and trypsin–EDTA were provided by Thermo Fisher Scientific (Waltham, MA, USA). A high-capacity RNA-to-cDNA kit was purchased from Thermo Fisher Scientific. The ReliaPrep RNA Cell Miniprep system, Caspase-Glo 3/7 assay, Caspase-Glo 9 assay, CellTiter-Glo luminescent cell-viability assay, and ROS-Glo H_2_O_2_ assay, were provided by Promega (Fitchburg, WI, USA). FBS Gold was from Thermo Fisher Scientific, a fluorescein isothiocyanate (FITC)–annexin V apoptosis-detection kit from BD Biosciences (San Jose, CA, USA), and radioimmunoprecipitation-assay lysis buffer and BCA protein-assay kit from Thermo Fisher Scientific. Molecular-grade purity water was provided by Sigma-Aldrich (St Louis, MO, USA). The polyclonal (mouse) anti-KDEL antibody was purchased from Enzo Biochem (Farmingdale, NY, USA). Horseradish (HRP)-conjugated anti-mouse IgG was from Rockland Immunochemicals (Limerick, PA, USA). HRP-conjugated anti-rabbit IgG, polyclonal (rabbit) anti-β-tubulin, monoclonal (rabbit) anti-CHOP, monoclonal (rabbit) anti-Casp 3 (cleaved), monoclonal (rabbit) anti-Casp 9 (cleaved), polyclonal (rabbit) anti-SOD1, and monoclonal (rabbit) anti-SOD2 antibodies were provided by Cell Signaling Technology (Boston, MA, USA). Quercetin (≥95% (HPLC), DMSO soluble ≥ 100 mg/mL) was purchased from Merck KGaA (Darmstadt, Germany).

### 4.2. Cell Culture

The human glioblastoma cell line A172 was purchased from American Type Culture Collection (ATCC), and LBC3 cell line was developed from *glioblastoma multiforme* tissue taken from 56-year-old female patient subjected to surgical tumor resection, and was kindly given to us by Prof. Cezary Marcinkiewicz (Department of Neuroscience, Temple University, Philadelphia, PA, USA) [65]. Cells were cultured in high-glucose DMEM with 10% of heat-inactivated FBS Gold, penicillin (100 U/mL), streptomycin (100 μg/mL), and 2 mmol/L l-glutamine. Cells were cultured in Falcon flasks (BD Biosciences) in a 5% CO_2_ incubator (Galaxy S+; Eppendorf, Hamburg, Germany) at 37 °C. Cells reaching subconfluence were detached from the culture plates using 0.05% trypsin 0.02%–EDTA in calcium-free PBS and counted in a Scepter cell counter (Merck Millipore, Billerica, MA, USA). Next, appropriate number of cells was seeded in DMEM 96- or 6-well plates. After 24 h of incubation, DMEM was removed and replaced with DMEM containing quercetin in the indicated concentrations. Control cells were treated with the vehicle-DMSO in the concentration of 0.25% in culture medium. Cells were then tested with the appropriate experimental protocols.

### 4.3. Cell Viability and QCT Cytotoxicity

The viability of A172 and LBC3 cells was evaluated according to methods in Kusaczuk et al. [66]. Briefly, cells were seeded in 96-well plates at a density of 1 × 10^3^ per well. Confluent cells were then cultured with QCT at concentrations of 5–400 μmol/L for 24 h and 48 h. Next, cells were washed twice with PBS and incubated with 1 mL MTT solution (0.25 mg/mL in PBS) at 37 °C in a humidified 5% CO_2_ atmosphere for 3 h. The medium was removed and formazan products solubilized in 1 mL of 0.1 mmol/L HCl in absolute isopropanol. Absorbance of a converted dye in living cells was read on a microplate reader (Tecan, Männedorf, Switzerland) at a wavelength of 570 nm. The viability of QCT-treated cells was calculated as a percentage of control untreated cells.

Additionally, in order to confirm MTT results, measurement of cellular ATP levels in control and QCT-treated A172 and LBC3 cells was performed using the CellTiter-Glo assay following the supplier’s specifications. Briefly, A172 and LBC3 cells were seeded in a white-walled 96-well culture plates (Nunclon, Thermo Fisher Scientific) at a density of 1 × 10^3^/per well. Cells were allowed to attach and then incubated with medium containing QCT in concentrations of 5–400 μmol/L at 37 °C for 24 and 48 h. After incubation, 100 μL staining solution (CellTiter-Glo reagent) was added to each well and mixed at 300 rpm on an orbital plate shaker for 2 min to induce cell lysis. Cells were incubated at room temperature for 10 min to stabilize the luminescence signal, which was recorded using the microplate reader.

### 4.4. Cell Morphological Analysis

To visualize morphological characteristics of glioblastoma A172 and LBC3 cells exposed to QCT treatment, cells were double-stained with acridine orange (AO) and ethidium bromide (EtBr). Staining was followed by fluorescence-microscopy observations. AO is able to enter both dead and viable cells. It emits red fluorescence when bound to single-stranded DNA, found predominantly in dead cells, and green fluorescence when bound to double-stranded DNA observed in viable cells. EtBr is actively excreted from living cells [67]. A172 and LBC3 cells at a density of 2.5 × 10^5^ were seeded into six-well plates and incubated with 50 and 100 μmol/L QCT at 37 °C in a humidified atmosphere containing 5% CO_2_ for 24 h and 48 h. After incubation, cells were stained with a mixture of AO (10 μg/mL) and EtBr (10 μg/mL). Cells were visualized using fluorescence microscope (CKX 41; Olympus, Tokyo, Japan) at 100× magnification.

### 4.5. Detection of Apoptosis and Necrosis by Flow Cytometry

Apoptosis of A172 and LBC3 cells was evaluated using the FITC–annexin V apoptosis-detection kit followed by flow-cytometry analysis. The cells were seeded in a 6-well plates at a density of 2.5 × 10^5^ per well (in 2 mL of medium) and cultured until they reached confluence. The cells were grown in high-glucose DMEM with 50 μmol/L and 100 μmol/L of QCT, for 24 h and 48 h. Next, cells were trypsinized and resuspended in DMEM and then in a binding buffer. The cells were stained using FITC–annexin V and propidium iodide (PI) for 15 min in the dark, according to the manufacturer’s manual. Flow cytometry analysis was performed using the FACSCanto II cytometer (BD FACSCanto II, San Diego, CA, USA). Analysis of data was performed using the FACSDiva software (BD, San Diego, CA, USA). The dead cells were discriminated on the basis of forward- and side-scatter parameters; annexin V+/PI- were identified as early apoptotic, annexin V+/PI+ as late apoptotic cells, and annexin V–/PI+ (Q1 quadrant) as necrotic cells. A sum of Q2 and Q4 quadrant populations of analyzed cells was presented as the percentage of apoptotic cells.

### 4.6. Caspase 3/7 and Caspase 9 Activities

Measurement of caspase 3/7 and caspase 9 activities after QCT treatment was performed using the luminescent Caspase-Glo 3/7 and Caspase-Glo 9 assays following the manufacturer’s instructions. Briefly, A172 and LBC3 cells were seeded in white-walled 96-well culture plates (Nunclon) at a density of 1 × 10^3^/well. Subsequently, cells were incubated with medium containing QCT at concentrations of 50 μmol/L and 100 μmol/L for 24 h and 48 h. After incubation, 100 μL Caspase-Glo 3/7 or Caspase-Glo 9 reagent was added to each sample. Cells were mixed using a plate shaker at 300 rpm for 45 s and left in the dark at room temperature for 40 min, followed by measurement of luminescence with a microplate reader (Tecan).

### 4.7. Reactive Oxygen-Species Generation

Generation of ROS was detected using the luminescent ROS-Glo H_2_O_2_ assay. A172 and LBC3 cells were plated at a density of 1 × 10^3^ per well in 80 μL DMEM in 96-well white-walled plates (Nunclon), as recommended by the manufacturer. Briefly, cells were allowed to attach to the plates at 37 °C in a CO_2_ incubator, and then growth medium was replaced with DMEM containing 50 μmol/L and 100 μmol/L QCT for 24 h and 48 h. Substrate solution was added to cells in a final concentration of 25 μmol/L. Then, cells were returned to the incubator (5% CO_2_, 37 °C) and cultured for 6 h. After this, 100 μL ROS-Glo detection solution was added to each well for 20 min at room temperature, and then relative luminescence units were recorded using the microplate reader.

### 4.8. RNA Isolation and Gene Expression Analysis

Total RNA was isolated using the ReliaPrep system with DNase I treatment according to the manufacturer’s instructions. Spectrophotometric measurements were performed to evaluate the quality and quantity of the extracted RNA (NanoPhotometer; Implen, Munich, Germany). Synthesis of cDNA was performed using the high-capacity RNA-to-cDNA Kit following the supplier’s recommendations. Briefly, 0.5 μg of purified total RNA was used in a 20 μL reaction mixture containing oligo(dT)16 primers, random octamers, dNTPs and murine leukemia virus reverse transcriptase (RT). cDNA (2 μL) served as a template for real-time RT quantitative polymerase chain reaction (qPCR). Amplification of the product was performed using 2×HS-PCR Master Mix SYBR A (A&A Biotechnology, Gdynia, Poland). Primer sequences for *Ddit3* (*Chop*), *Bax*, *Puma*, *Noxa*, *Hspa5* (*Grp78*), *Sod1*, *Sod2*, *Cat* and housekeeping *Rpl13a* have been described in our previous works [43,66,68]. Additional evaluation of primer accuracy was carried out using Primer-BLAST software. The following reaction parameters were applied: initial denaturation at 95 °C for 3 min, followed by 40 cycles of 95 °C for 1 min, 60–63 °C for 30 s, and 72 °C for 45 s. The CFX Connect real-time PCR system (Bio-Rad Laboratories, Hercules, CA, USA) was used to perform a real-time qPCR assay. Reactions were run in triplicates and expressions were analyzed using the relative quantification method modified by Pfaffl [69].

### 4.9. Protein Assay

A172 and LBC3 cells were seeded in six-well plates and treated as previously described. After treatment, cells were washed with cold PBS and solubilized in 100 μL radioimmunoprecipitation-assay lysis buffer per well. Cell lysates were then subjected to centrifugation (14,000× *g* at 4 °C for 10 min), and supernatants were collected for protein evaluation. The BCA protein-assay kit was used to determine protein concentration in cell lysates. Protein assays were performed according to the method described in our previous works [43,66]. BSA was used as a standard.

### 4.10. Sodium Dodecyl Sulfate–Polyacrylamide-Gel Electrophoresis

Samples of the lysates containing 10 μg of protein were subjected to sodium dodecyl sulfate–polyacrylamide-gel electrophoresis as described in our previous works [43,66]. Electrophoresis was run for 40–45 min using a 10–12% polyacrylamide-gel. A constant current of 25 mA was applied.

### 4.11. Immunoblotting

Resolved proteins were transferred to polyvinylidene difluoride (PVDF) membranes and preincubated with Tris-buffered saline (TBS) containing 0.05% Tween 20 (TBS-T) and 5% nonfat dry milk for 2 h. Membranes were soaked in a mixture of anti-KDEL (1:1000), anti-CHOP (1:1000), anti-Casp 3 (cleaved) (1:1000), anti-Casp 9 (cleaved) (1:1000), anti-SOD1 (1:1000), anti-SOD2 (1:1000), and anti-β-tubulin (1:1000) antibodies in 5% dried milk in TBS-T at 4 °C for 16 h. Next, 1-h incubation with secondary HRP -conjugated antibody against mouse or rabbit IgG at 1:2500 dilution was carried out. Finally, the PVDF membranes were washed five times with TBS-T and exposed to SignalFire Elite ECL Reagent (Cell Signaling). Images were visualized using GeneGnome XRQ Chemiluminescence system (Syngen, Cambridge, UK).

### 4.12. Zeta Potential Analysis

Electrophoretic mobility of cell membrane was carried out using the electrophoretic light scattering technique on Zetasizer Nano ZS analyzer equipped with a 4 mW He-Ne laser (Malvern Instruments, Malvern, UK). The measurements were performed as a function of pH (in pH range 2–10). Briefly, A172 and LBC3 cells were suspended in 0.9% NaCl and titrated to the desired pH with HCl or NaOH. The reported values represent the average of six measurements at a given pH value. Based on electrophoretic mobility values, the surface charge density (*δ*) was determined from the Equation (1):

(1) $$\delta =\frac{\eta \cdot u}{d}$$

in which *η* is the viscosity of solution; *u* the electrophoretic mobility; and *d* the diffuse layer thickness.

### 4.13. Statistical Analysis

Results are presented as mean ± SD from three independent experiments run in triplicate. GraphPad Prism 5 software (GraphPad Software, Inc., USA) was applied for statistical analysis. One-way analysis of variance was carried out for comparisons between control and treated groups. The half maximal inhibitory concentration (IC_50_) values were calculated using the GraphPad Prism 5 software. Pairwise comparisons were made by post hoc Tukey’s test. Differences were considered significant for *p* < 0.05.

## 5. Conclusions

The utilization of natural substances, such as plant polyphenols, as potential cytostatic agents represents a strong trend in current oncopharmacology. In this study we demonstrated that quercetin might potentially be considered as an anti-glioblastoma agent due to its pro-apoptotic activity in GBM cells. However, these results need to be treated with proper caution regarding necrotic cell death occurring after prolonged exposition to QCT in LBC3 cell line. In this respect, further investigations need to be carried out to fully explore the mechanisms activated after QCT exposure in a wide variety of GBM cell lines to get a full spectrum of possible molecular effects of this polyphenol in brain tumor cells.

## Figures and Tables

**Table 1:** The influence of QCT on selected human glioblastoma cell lines showing various mutation status in main tumor progression-related genes. *Idh1*-isocitrate dehydrogenase (NADP(+)) 1; *Pten*-phosphatase and tensin homolog deleted on chromosome 10; mut-mutant; wt-wild-type.

Human GBM Cell Lline	Gene Mutation Status	Tumori-Genicity	Reported QCT Effect	References
T98G	*p53* (mut) *Pten* (mut) *Idh1* (wt) *p16* (mut)	-	-inhibition of JAK/STAT3 pathway -inhibition of cyclin D1 expression -activation of, caspase 3 and 9 -cytochrome c release -suppression of cel growth and migration -inhibition of autophygy -induction of apoptosis	[11,16,20,22,26,27]
U87	*p53* (wt) *Pten* (mut) *Idh1* (wt) *p16* (mut)	+	-inhibition of Hsp27 expression -sensitization to temozolomide treatment -degradation of survivin -inhibition of the Ras/MAPK/ERK and PI3K/AKT signalling pathways	[19,20,23,27,28,29]
U251	*p53* (mut) *Pten* (mut) *Idh1* (wt) *p16* (mut)	-	-sensitization to temozolomide treatment -reduction in the phospho-AKT level -increased expression of p53 -degradation of survivin -inhibition of the Ras/MAPK/ERK and PI3K/AKT signalling pathways -increased cleavage of caspase-3, caspase-9 and PARP-1 -induction of apoptosis and autophagy	[13,15,19,20,23,27,30,31]
A172	*p53* (mut) *Pten* (mut) *Idh1* (wt) *p16* (mut)	-	-degradation of survivin -depolarization of mitochondrial membrane potential -downregulation of ERK, AKT, and survivin -activation of caspase-3	[17,27,29,32,33]
LBC3	*p53* (mut) *Pten* (wt)	-not tested	-not tested	[34]

## Data Availability

The data presented in this study are available within the manuscript.
